# Dupilumab for the treatment of prurigo nodularis: A systematic review

**DOI:** 10.3389/fimmu.2023.1092685

**Published:** 2023-01-20

**Authors:** Peng Cao, Wenjing Xu, Shuyi Jiang, Litao Zhang

**Affiliations:** ^1^ Graduate School, Tianjin Medical University, Tianjin, China; ^2^ Department of Dermatology, Tianjin Academy of Traditional Chinese Medicine Affiliated Hospital, Tianjin, China; ^3^ Graduate School, Tianjin University of Traditional Chinese Medicine, Tianjin, China

**Keywords:** dupilumab, biologics, prurigo nodularis, itch, treatment

## Abstract

**Background:**

Conventional treatment techniques have limited efficacy and more side effects in the treatment of prurigo nodularis. The better alternative treatment option for better outcomes of the disease is dupilumab.

**Objective:**

The objective of this study was to systematically review dupilumab-related treatment outcomes in prurigo nodularis.

**Methods:**

Several databases like Embase, PubMed, Web of Science, and Cochrane library were searched for data acquisition on October 8, 2022. Based on Preferred Reporting Items for Systematic Reviews and Meta-analyses guidelines, 24 publications were included in this study.

**Results:**

After 4,12,16 and more than 16 weeks of dupilumab treatment, 8.3% (n=5/60), 34.4% (n=11/32), 3.6% (n=2/56), and 45.3% (n=29/64) of patients had complete remission, respectively. In addition, 85.0% (n=51/60), 59.4% (n=19/32), 83.9% (n=47/56), and 43.8% (n=28/64) had partial remission, respectively. Moreover, 6.7% (n=4/60), 6.3% (n=2/32), 12.5% (n=7/56), and 10.9% (n=7/64) showed no remission, respectively, and significant reduction of numeric rating scale itch intensity (from 9.0 to 4.9, 2.1, 2.8, 0.9) was attained. There were no serious adverse events observed during treatment, but the most common event observed was conjunctivitis (12.6%, n=15/119).

**Conclusions:**

Dupilumab has definite effectiveness and safety in prurigo nodularis treatment.

**Systematic review registration:**

https://www.crd.york.ac.uk/PROSPERO, identifier (CRD42022365802).

## 1 Introduction

Prurigo nodularis (PN) is a chronic inflammatory skin disease characterized by persistent isolated pruritic firm papules and nodules. PN might be associated with a variety of systemic diseases such as chronic nephritis, type 2 diabetes, and human immunodeficiency virus infection. PN was most commonly observed in middle-aged people and more prevalent in females than in males. It has an estimated prevalence rate of 72/100,000 individuals in the age range of 18-64 (1, 2). While the pathogenesis of PN is still unclear, it may be associated with chronic itch followed by repeated scratching forming a vicious cycle, a process in which significant abnormal interactions and dysregulation between immune cells and neural circuits play an important role (3, 4).

The treatment goals of PN were to block the itch-scratch vicious cycle, reduce itching and eliminate lesions (5, 6). Traditional treatments include topical corticosteroids, topical calcineurin inhibitors, topical capsaicin, gabapentinoids, antidepressants, opioid antagonists, immunosuppressants, and phototherapy, which have limited efficacy, accompanied by varying degrees of side effects (3, 6).

A human monoclonal IgG4 antibody called dupilumab is a dual inhibitor of the signaling of interleukin (IL)-4 and IL-13 by specifically binding to the IL-4 Rα subunit shared by the IL-4 and IL-13 receptor complexes. Dupilumab has demonstrated good efficacy and safety in a variety of diseases with type 2 inflammation like atopic dermatitis and asthma, it is now clear that the Th2 axis-related cytokines IL-4 and IL-13 are involved in the pathogenesis of PN (7, 8). Dupilumab has been approved by the Food and Drug Administration (FDA) for the treatment of PN in adults (9), and a series of small retrospective studies and case reports suggested its safety and effectiveness in the treatment of PN. Therefore, the aim of this study was to systematically assess and evaluate the reports available on dupilumab usage and its outcomes in PN patients.

## 2 Methods

### 2.1 Search strategy and eligibility

On October 8, 2022, following the Preferred Reporting Items for Systematic Reviews and Meta-Analyses guidelines and PROSPERO international prospective registry (CRD42022365802), a systematic review was performed, and registered articles were obtained from databases like Cochrane library, Embase, PubMed, and Web of science (10). The search string used for combining Medical Subject Headings (MeSH) terms and keywords were as follows: ((dupilumab) OR (((((SAR231893) OR (SAR-231893)) OR (Dupixent)) OR (REGN668)) OR (REGN-668))) AND ((prurigo) AND ((nodularis) OR (nodular))). Vocabulary and syntax were adapted to be appropriate for each database. Moreover, the authors manually searched the references given in the reviewed articles to identify in an independent manner those that may have been missed by the search engines.

Only those articles that documented PN-based patient diagnosis and reported outcomes with dupilumab treatment against PN were included in this study.

### 2.2 Outcomes

The remission outcomes of dupilumab were as follows: (1) Complete remission: Numeric rating scale itch intensity (NRSI) reduced to 0 at follow-up. The terms used for these outcomes in the reference publications were as follows: “complete remission/resolution/response/control” or “symptom-free”. (2) Partial remission: At follow-up, NRSI decreased compared to baseline levels, but remained greater than 0. The terms used in the reference articles were as follows: “partial remission/resolution/response/control”, “improved” or “clinical improvement”. (3) No remission: NRSI did not decrease at follow-up compared to baseline levels. The terms used were as follows: “no remission/resolution/response/control” or “deterioration”.

NRSI: NRSI was graded from 0 (no itch) to 10 (insupportable itching). Baseline NRSI prior to dupilumab initiation and NRSI at each subsequent visit after dupilumab initiation were recorded.

Adverse events: Dupilumab-related adverse events such as conjunctivitis and injection site reaction during treatment have been recorded.

### 2.3 Data screening and selection

The articles that were selected in the screening strategy were reviewed initially on the basis of titles and abstracts by the two authors (Cao and Xu) independently, followed by the investigation of the full text of potentially eligible studies. If there were any disagreements regarding data in the articles between two authors, they were resolved through discussion with a third author (Zhang).

For each selected study, the following information will be extracted into an electronic form: first author, year of publication, design of the study, number of patients, gender, age, PN onset age, PN duration, personal atopic history, family history of AD, previous treatment, NRSI, adverse events, and resolution outcomes (complete remission, partial remission, no remission).

### 2.4 Quality assessment

The data were evaluated for quality assurance in accordance with the Oxford Centre for Evidence-Based Medicine: Levels of Evidence (2009).

### 2.5 Statistical analyses

Data were analyzed using descriptive statistics, and statistical graphs were plotted by GraphPad prism 9.0. Numbers and percentages were used for representing categorical variables, whereas mean and range were used for representing continuous variables.

## 3 Results

**Figure 1:**
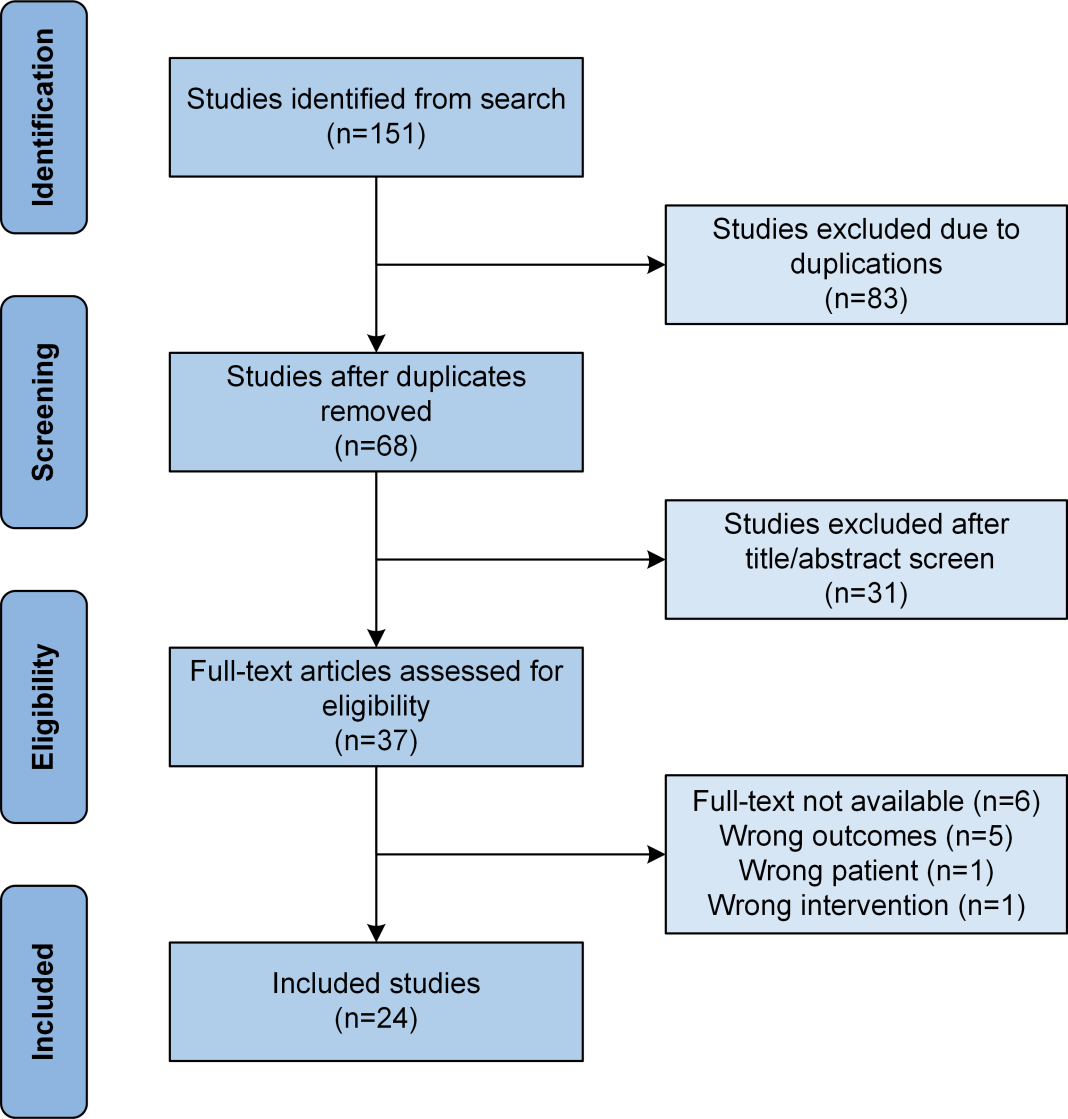
Flow chart showing study selection based on the Preferred Reporting Items for Systematic Reviews and Meta-Analyses (PRISMA) guidelines.

A total of 151 publications were retrieved from the databases and further reviewed for inclusion and exclusion. Initially, 83 articles were excluded because of duplication, followed by the exclusion of 31 more articles once their titles and abstracts were reviewed. The other 13 articles were excluded when they were further studied, leaving behind only 24 articles that fulfilled the selection criteria and were included in the study (**Figure 1**). The 24 publications included only 1 prospective study (11), 5 retrospective studies (12–16), 5 case series (17–21), and 13 case reports (22–34).

The total number of patients that were included in the study was 119, as shown in **Table 1**. The mean age calculated for these patients was 53.9 years (ranging from 9.0 years to 87.0 years). Gender distribution revealed that 57.1% (n=68/119) of the participants were female, 40.3% (n=48/119) were male, and 2.5% (n=3/119) had no gender information given. The mean age of PN onset calculated was 45.8 years (ranging from 6.0 to 83.0 years), with the mean disease duration of 10.3 years (ranging from 2.0 to 27.0 years). The percentage of patients with a history of atopic dermatitis (AD) or AD manifestations was 47.1% (n=56/119); followed by 28.6% (n=34/119) with allergic rhinitis, 18.5% (n=22/119) with asthma, 9.2% (n=11/119) with allergic conjunctivitis, and 0.8% (n=1/119) with urticaria. The other 18.5% (n=22/119) had no personal atopic history, and 6.7% (n=8/119) had no reports given. While 18.5% (n=22/119) had a family history of AD, 37.8% (n=45/119) had no family history of AD, and 43.7% (n=52/119) had no reports given.

**Table 1:** Summary of baseline characteristic and demographic information in patients with prurigo nodularis.

Demographics	Value
Patients, n (%)	119 (100.0)
Sex, n (%)	
Female	68 (57.1)
Male	48 (40.3)
NR	3 (2.5)
Age, y	
Mean	53.9
Range	9.0-87.0
NR, n (%)	11 (9.2)
PN onset Age, y	
Mean	45.8
Range	6.0-83.0
NR, n (%)	42 (35.3)
PN duration, y	
Mean	10.3
Range	2.0-27.0
NR, n (%)	58 (48.7)
Personal atopic history, n (%)	
AD or AD manifestations	56 (47.1)
Allergic rhinitis	34 (28.6)
Asthma	22 (18.5)
Allergic conjunctivitis	11 (9.2)
Urticaria	1 (0.8)
None	22 (18.5)
NR	8 (6.7)
Family history of AD, n (%)	
Yes	22 (18.5)
No	45 (37.8)
NR	52 (43.7)
Previous treatment, n (%)	
Failed one systemics treatment	91 (76.5)
Failed three or more systemics treatment	65 (54.6)
Topical corticosteroids	77 (64.7)
Phototherapy	54 (45.4)
Systematic corticosteroids	48 (40.3)
Antihistamines	39 (32.8)
Thalidomide	10 (8.4)
Gabapentinoids	8 (6.7)
Antibiotics	6 (5.0)
Cryotherapy	5 (4.2)
Systemic retinoids	5 (4.2)
Dronabinol	2 (1.7)
Lenalidomide	1 (0.8)
Apremilast	1 (0.8)
Cannabidiol	1 (0.8)
Immunosuppressants	
Cyclosporine	62 (52.1)
Methotrexate	44 (37.0)
Azathioprine	17 (14.3)
Tacrolimus	17 (14.3)
Mycophenolate mofetil	8 (6.7)
Pimecrolimus	1 (0.8)
Antidepressants	
Mirtazapine	6 (5.0)
Doxepin	3 (2.5)
Paroxetine	1 (0.8)
Amitriptyline	1 (0.8)
Opioid antagonists	
Naltrexone	3 (2.5)
Naloxone	1 (0.8)
None	0 (0.0)
NR	10 (8.4)

All patients had been previously treated with at least 1 treatment strategy but failed, and 76.5% (n=91/119) and 54.6 (n=65/119) failed at least 1,3 systemic treatments before dupilumab therapy, respectively. Before dupilumab treatment, 64.7% (n=77/119), 45.4% (n=54/119), 40.3% (n=48/119), 32.8% (n=39/119), 8.4% (n=10/119), 6.7% (n=8/119), 5.0% (n=6/119), 4.2% (n=5/119), 4.2% (n=5/119), 1.7% (n=2/119), 0.8% (n=1/119), 0.8% (n=1/119), 0.8% (n=1/119) received topical corticosteroids, phototherapy, systematic corticosteroids, antihistamines, thalidomide, gabapentinoids, antibiotics, cryotherapy, systemic retinoids, dronabinol, lenalidomide, apremilast and cannabidiol respectively but failed; 52.1% (n=62/119), 37.0% (n=44/119), 14.3% (n=17/119), 14.3% (n=17/119), 6.7% (n=8/119), 0.8% (n=1/119) received immunosuppressants cyclosporine, methotrexate, azathioprine, tacrolimus, mycophenolate mofetil and pimecrolimus respectively but failed; 5.0% (n=6/119), 2.5% (n=3/119), 0.8% (n=1/119), 0.8% (n=1/119) received antidepressants mirtazapine, doxepin, paroxetine and amitriptyline respectively but failed; 2.5% (n=3/119), 0.8% (n=1/119) received opioid antagonists naltrexone and naloxone respectively but failed.

**Figure 2:**
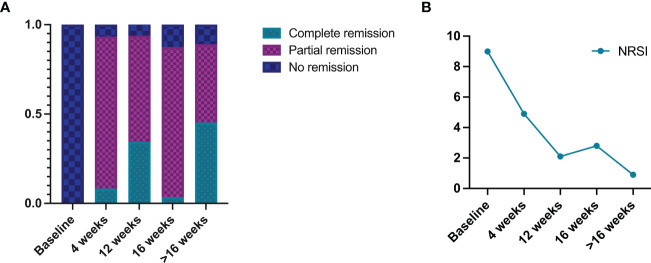
Summary of dupilumab treatment outcomes in prurigo nodularis patients: **(A)** resolution outcomes, **(B)** numeric rating scale itch intensity (NRSI).

Complete remission was observed in 8.3% (n=5/60) of patients after 4 weeks of dupilumab treatment, 85.0% (n=51/60) attained partial remission, and 6.7% (n=4/60) showed no remission (**Figure 2A**). Moreover, a significant reduction of NRSI (from 9.0 to 4.9) was attained (**Figure 2B**).

After 12 weeks of dupilumab treatment, complete remission was observed in 34.4% (n=11/32) patients, 59.4% (n=19/32) attained partial remission, and 6.3% (n=2/32) showed no remission. Moreover, a significant reduction of NRSI (from 9.0 to 2.1) was attained.

After 16 weeks of dupilumab treatment, 3.6% (n=2/56) of patients had complete remission, 83.9% (n=47/56) had partial remission, and 12.5% (n=7/56) showed no remission. Moreover, a significant reduction of NRSI (from 9.0 to 2.8) was attained.

After more than 16 weeks of dupilumab treatment, 45.3% (n=29/64) of patients had complete remission, 43.8% (n=28/64) attained partial remission, and 10.9% (n=7/64) showed no remission. Moreover, a significant reduction of NRSI (from 9.0 to 0.9) was attained.

**Figure 3:**
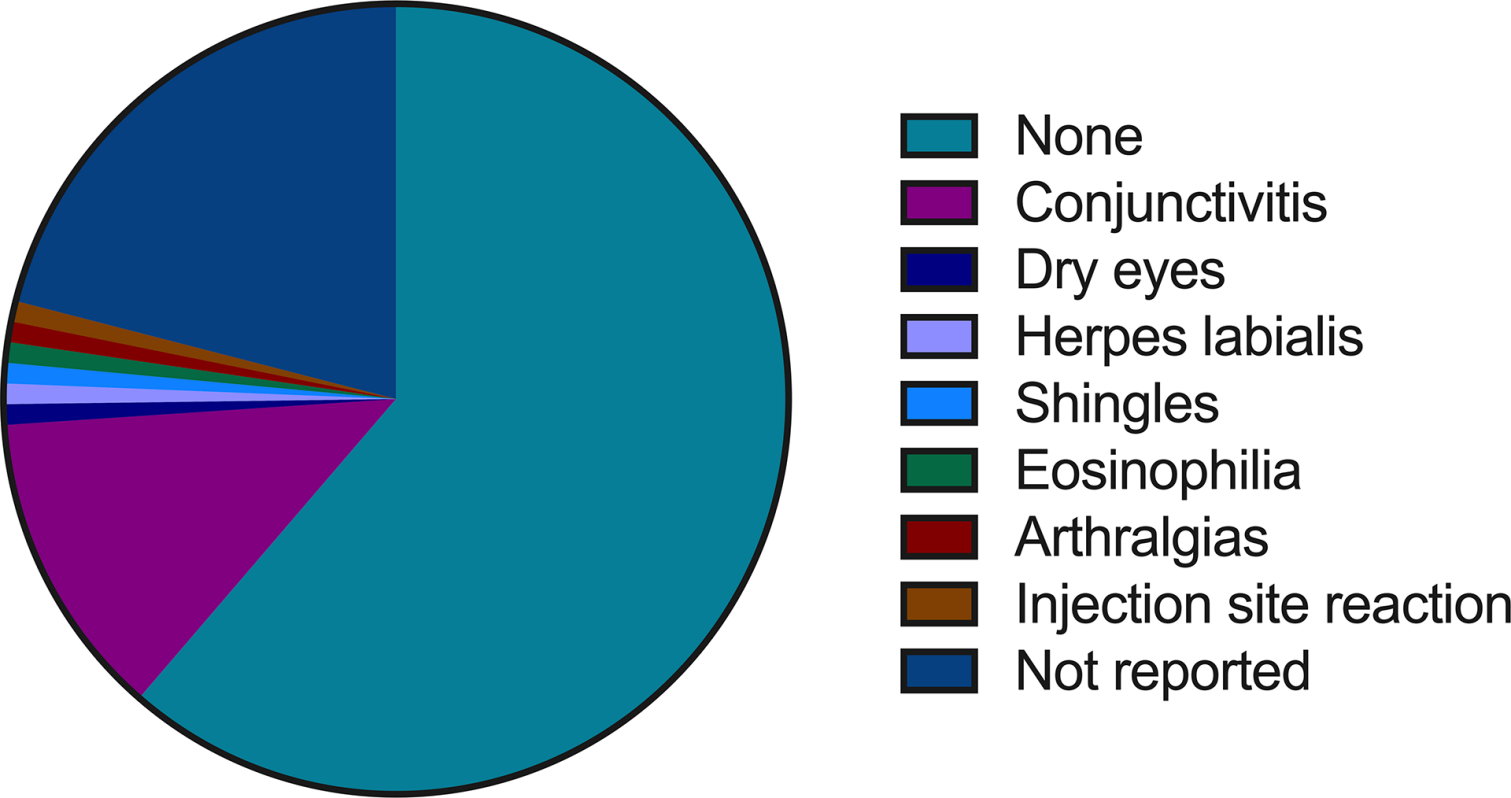
Summary of dupilumab treatment outcomes in prurigo nodularis patients: adverse events.

There were no adverse events reported in 61.3% (n=73/119) of patients (**Figure 3**), whereas 21.0% (n=25/119) of the total patients had no information available related to adverse events. Some patients showed different (slight) adverse events like conjunctivitis (12.6%, n=15/119), which was reported to be the most common of all adverse events, followed by dry eyes (0.8%, n=1/119), herpes labialis (0.8%, n=1/119), shingles (0.8%, n=1/119), eosinophilia (0.8%, n=1/119), arthralgias (0.8%, n=1/119), and injection site reaction (0.8%, n=1/119).

## 4 Discussion

Dupilumab showed definite effectiveness in the treatment of PN. These patients had significantly lower NRSI at 4, 12, 16, and more than 16 weeks follow-up, and at least 87.5% of PN patients achieved clinical remission. A systematic review by Husein et al. showed a reduction in pruritus in 82.2% of PN patients after treatment with dupilumab (35). Both the recent phase III trials reported 58% and 60% of PN patients treated with dupilumab achieved clinical remission, respectively, a figure below 87.5%. This could be because the vast majority of publications included in this systematic review were case reports (54.2%, n=13/24) and case series (20.8%, n=5/24), and seldom cases regarding the ineffectiveness of dupilumab in treating PN were reported.

The reduction of NRSI from 9.0 to 4.9 in PN patients at 4 weeks of dupilumab treatment and the achievement of clinical remission in 93.3% (n=56/60) of patients suggested that PN patients might have responded to dupilumab earlier. 93.3%, 93.8% and 87.5% clinical remission rate at 4, 12 and 16 weeks of treatment, respectively. However, NRSI was further reduced at 12 and 16 weeks of treatment compared to 4 weeks, suggesting that the longer treatment cycles within a certain range contribute to a greater reduction in pruritus symptoms. Therefore, it is crucial for those PN patients whose initial treatment with dupilumab was effective to complete 16 weeks or at least 12 weeks of treatment. By more than 16 weeks of treatment, the NRSI decreased to 0.9, clinical remission rates continued to remain stable, and complete remission rates significantly increased compared to 4,12, and 16 weeks of treatment. In one of the previously reported studies by Jorge et al., the proportion of patients achieving investigator’s global assessment (IGA) 0/1 at 52 weeks of dupilumab treatment was higher than the proportion of patients achieving IGA 0/1 at 16 weeks of dupilumab (14). It was suggested that PN patients who completed their 16 weeks of dupilumab treatment but still did not achieve complete remission could extend the treatment cycle further to achieve complete remission. However, patients who remained ineffective to dupilumab treatment for 16 weeks were not recommended to continue extended treatment cycles to seek clinical remission. This delayed response was uncommon and only a very small percentage of PN patients who remained ineffective on dupilumab for 16 weeks could achieve clinical remission with extended treatment cycles (14). According to Husein et al., complete remission can be expected when a patient begins to notice any improvement (Time_First) which is nearly 8 weeks following dupilumab treatment and the NRS has decreased by 50% (NRS_50). In contrast, the complete response will not likely happen if the Time_First is 12 weeks or longer, and the NRS_50 is not reached (35).

There is no clear mechanism by which dupilumab treatment of PN achieves clinical remission. IL-4, signal transducer and activator of transcription (STAT) 6 expression was upregulated in skin lesions of PN patients (36). IL-4 and IL-13 have been shown to play a role in the direct activation of Th2 inflammatory factor receptors in sensory neurons in the dorsal root ganglia, resulting in intense itching (37). A mouse model of chronic pruritus suggests that IL-4 and IL-13 activate itch-sensory neuronal pathways *via* IL-4Rα receptor (type I and type II), IL-13 Rα1 receptor (type II receptor) and Janus kinase 1 signaling (37). Dupilumab inhibits IL-4 signaling by binding to type I and II receptors and IL-13 signaling by binding to type II receptors, which in turn blocks the Th2 cell-mediated inflammatory responses resulting in the blockage of significant abnormal interactions and dysfunction between immune cells and neural circuits driving the pruritic-scratch cycle, thus prompting the PN patients to achieve clinical remission (7, 12).

Dupilumab showed an established safety profile for PN, as there were no patient deaths and no serious adverse events during treatment, and 61.3% (n=73/119) of PN patients showed no adverse events. Some slight adverse events observed were all subsided with the treatment with no interruption to dupilumab therapy. The most common adverse event was conjunctivitis (12.6%, n=15/119), which was slightly higher than conjunctivitis observed in AD patients treated with dupilumab (12.6% vs. 8.3%) reported by Simpson et al. (38 *)*,. It is still unclear about the mechanism by which dupilumab induces conjunctivitis. However, it might be due to dupilumab’s blockade of the IL-4 and IL-13 signaling pathways, which leads to the decrease in conjunctival cupped cell and mucin secretion, leading to tear film instability and mucosal epithelial barrier dysfunction that ultimately leads to conjunctival inflammation, but this process is reversible when patients discontinue dupilumab (39, 40). Moreover, it may be beneficial to use artificial tears prophylactically at the start of dupilumab treatment to reduce the incidence of conjunctivitis (41, 42). Eosinophils were involved in the pathogenesis of AD and PN. In patients with moderate-to-severe AD treated with dupilumab, Absolute Eosinophil Counts (AEC) gradually increased after dupilumab treatment, which reached a peak at 4 months, then decreased and returned to the baseline level at 12 months. AEC in moderate-to-severe AD patients with dupilumab-related ocular surface disease or facial redness dermatitis increased greatly at 4 months, so monitoring ACE in AD and PN patients during dupilumab treatment may predict the risk of developing dupilumab-related adverse events (43).

There were two patients with PN combined with breast cancer, chronic lymphocytic leukemia, and pancreatic intraductal papillary mucinous tumor, respectively, included in this systematic review. Both the patients experienced clinical remission with PN after dupilumab treatment, with no serious adverse events and no tumor recurrence or progression occurred. It was reported in previous studies that three patients who had AD combined with melanoma, squamous cell carcinoma of the rectum, and non-Hodgkin’s lymphoma, respectively, treated with dupilumab achieved complete remission without serious adverse events and did not cause tumor recurrence or progression (44, 45). This systematic review included one PN patient living with HIV who achieved clinical remission after dupilumab treatment without serious adverse events, also whose HIV viral load and CD4 counts remained stable during treatment and follow-up. A case report and literature review showed that dupilumab was safe and effective in treating AD patients living with HIV, and without negatively impacting HIV control (46). Some of the PN patients included in this systematic review also had comorbid systemic diseases such as diabetes mellitus, and cerebrovascular and cardiovascular diseases. These patients achieved clinical remission, and no serious adverse events were observed after dupilumab treatment. Dupilumab has been shown to be safer and more effective in controlling patients with PN in combination with other underlying diseases, even HIV infection or malignancies. Dupilumab’s long-term safety data in patients with systemic diseases like cardiovascular disease, HIV infection and malignancies remain insufficient, and further studies are required.

There were two pediatric patients aged nine and sixteen years included in this systematic review, and both achieved partial remission of PN after treatment with dupilumab with no adverse events. Dupilumab has not been approved by the FDA for the treatment of PN in children. Nonetheless, dupilumab has been widely used since its approval for the indication of AD in children, and it has shown definite efficacy and safety. Although dupilumab has the potential to be a safer and more effective treatment for PN in children, it needs to be supported by more data.

This study’s limitations include small sample size and a lack of a control group. Another limitation is publication bias, which occurs when research with negative results is less likely to be published. The underlying data included in this systematic review are subject to publication bias consisting of case report (54.2%, n=13/24), case series (20.8%, n=5/24), retrospective (20.8%, n=5/24), and small-size prospective studies (4.2%, n=1/24). Besides, the presence of AD or other atopic diseases may affect the outcomes of dupilumab treatment. We have access to information on the proportion of patients with comorbid AD or other atopic diseases, but we are unable to specify the individual atopic history of a specific patient, much less link it to treatment outcomes, and therefore we are unable to perform subgroup analyses based on the presence or absence of comorbid AD or other atopic diseases. In addition, the efficacy of dupilumab in the treatment of PN was slightly simple depending on NRSI alone, and the reduction of skin lesions was another effective indicator. However, due to the limitation that most studies included in this systematic review did not quantify the reduction of skin lesions in patients, and it was only a general description, so this systematic review did not take the reduction of skin lesions as an outcome indicator.

## 5 Conclusion

This systematic review thoroughly summarizes the published data of dupilumab for PN treatment to date. Our study demonstrated the definite effectiveness and dupilumab’s safety in PN treatment. In the future, more clinical trials are required to determine the efficacy and safety of dupilumab in PN patients.

## Data availability statement

The original contributions presented in the study are included in the article/supplementary material. Further inquiries can be directed to the corresponding author.

## Author contributions

PC, WX, and SJ: conception and design of the work, data collection, data analysis and interpretation, and manuscript drafting. LZ: conception and design of the work, data collection, data analysis and interpretation, article revision, and approval of the publication. All authors contributed to the article and approved the submitted version.
